# Design of Carbon Nanocomposites Based on Sodium Alginate/Chitosan Reinforced with Graphene Oxide and Carbon Nanotubes

**DOI:** 10.3390/polym15040925

**Published:** 2023-02-13

**Authors:** Gohar Khachatryan, Karen Khachatryan, Joanna Szczepankowska, Marcel Krzan, Magdalena Krystyjan

**Affiliations:** 1 Faculty of Food Technology, University of Agriculture in Krakow, Al. Mickiewicza 21, 31-120 Krakow, Poland; 2 Faculty of Biotechnology and Horticulture, University of Agriculture in Krakow, Al. Mickiewicza 21, 31-120 Krakow, Poland; 3 Jerzy Haber Institute of Catalysis and Surface Chemistry, Polish Academy of Sciences, 31-120 Krakow, Poland

**Keywords:** carbon, graphene oxide, carbon nanotubes, chitosan, sodium alginate, nanocomposites

## Abstract

The aim of this study was to use a simple, low-cost and environmentally friendly synthesis method to design nanocomposites. For this purpose, carbon nanostructures were used to reinforce the chitosan/alginate bond in order to improve the mechanical, solubility, water absorption and barrier (protection against UV radiation) properties of the chitosan/alginate structure. Scanning electron microscopy (SEM), Fourier-transform infrared spectroscopy (FTIR), ultraviolet and visible light absorption spectroscopy (UV-VIS) and color analysis were utilized, and the thickness and mechanical properties of the obtained films were determined. The tests that were carried out showed an equal distribution of nanostructures in the composite material and the absence of chemical interactions between nanoparticles and polymers. It was also proven that the enrichment of the polysaccharide composite with graphene oxide and carbon nanotubes positively affected its absorption, mechanical capabilities and color.

## 1. Introduction

Chitosan is one of the most widely available polysaccharides in the world. Chitosan contains three types of reactive functional groups: an amine group, an acetamide group and primary and secondary hydroxyl groups located at the second, third and sixth carbon atoms. The amine group is the most important. It influences differences in physicochemical properties, such as solubility and chelation ability. Other characteristics of chitosan, which include viscosity, mechanical ability, adsorption on solids and biofunctional activity, depend on many other factors. These include the chitosan structure and degree of deacylation (affecting, among other things, viscosity—the unbranched and linear form with a high degree of deacylation shows better viscosity), crystallinity, degradation methods, ionization range and molecular weight [1,2,3,4,5,6,7]. In addition to the properties typical of all polysaccharides, chitosan is characterized by a wide range of unique biological properties. Of great importance, especially in medical science, are its bactericidal, bacteriostatic and antifungal abilities. Due to its cationic nature, it is able to bind to the negatively charged surface of bacteria and fungi and cause leakage of the cytoplasm, thus leading to cell death [3]. Fungal chitosan is successfully used in medicine as a membrane in wound dressings because of its wound-healing properties [5,6]. In the food industry, on the other hand, due to its coating properties and ability to store water, it can be used in the production of active packaging or as a food additive [2,4,6,8].

Alginate is a natural, biostable and hydrophilic polymer extracted on a large scale from marine algae. Alginate is completely biocompatible and has many promising physicochemical properties. The most important of these is its high ability to form hydrogels and retain moisture. It is capable of absorbing up to 300 times more water than it weighs by itself. For these reasons, it is commonly used as a stabilizer, thickener, emulsifier and gelling agent [9,10,11,12,13]. The characteristics of alginate promote its use in biomedical sciences. Due to its ability to maintain a humid environment and optimal pH, it is able to accelerate wound healing and tissue regeneration. For this reason, conventional dressings are increasingly being replaced by alginate-based dressings [10]. In addition, it is applied as a drug-coating element, facilitating its controlled release into selected tissues. It also displays the ability to improve insulin resistance, relieve inflammation and reduce oxidative stress. It can be used as an insulin-delivery agent. For these reasons, it is a potential tool in the treatment of diabetes. It has positive effects on the cardiovascular system. Many studies have shown its potential to lower cholesterol and blood glucose [9,14,15,16]. Alginate-based films are used to coat food products. They provide a good barrier, preventing the loss of volatile flavor compounds and protecting against microorganisms and viruses. Alginate can also be introduced into food in the form of nanocapsules containing various substances with preservative, antioxidant and color properties. This makes it possible to extend the shelf life of the product and improve its appearance and attractiveness [9,17,18].

Carbon nanoparticles are most often used as nanofillers in composite materials, as this combination can significantly increase their functionality. Their most important application properties include good electrical and thermal conductivity, electrochemical and thermal stability and mechanical strength. The latest reports have shown that carbon nanoparticles have been used as nanofillers. Polymer composites, after enriching with nanofillers, gain new features that allow them to be used as so-called “active,” “smart” and “enhanced” [19,20,21]. Recent studies have also demonstrated their high biocompatibility with cells such as osteocytes and neurons, as well as their antimicrobial activity, which greatly enhances their applicability in biomedical sciences [19,22,23,24,25].

The development of modern technologies influences the mobility of producing innovative materials characterized by high functionality. Great potential is shown by polymer nanocomposites, which are obtained by dispersing modifiers with dimensions of several nanometers in a polymer matrix. Their attractiveness is due to the fact that the matrix interacts with the dispersed phase already at the molecular level, which means that the nanofiller can significantly affect the selected parameters of the composite. The introduction of even a small amount of nano-additives is capable of significantly improving the mechanical, optical, electrical, thermal and bacteriostatic properties of the composite material [19,26,27,28]. Of particular interest are films that are mixtures of polysaccharides. They are classified as so-called biocomposites, i.e., composites in which at least one component is biobased or biodegradable. They are a good alternative to commonly used synthetic plastics, which are a heavy burden on the environment. Due to their easy availability and low production costs, they have great potential for applications in the industrial sector. In addition, the diversity of functional groups makes them an interesting material for modification [29,30,31,32,33]. Among the polysaccharides used in the production of composites, chitosan and alginate have gained considerable attention. In addition to the many favorable characteristics of all polysaccharide polymers, they exhibit a number of unique physicochemical and functional properties. This gives great opportunities to their applications in almost all areas of science and industry. However, despite their many advantages, polysaccharide films also have certain limitations. These are primarily poor mechanical, thermal and barrier properties. Therefore, in order for them to compete with currently used synthetic materials, it is necessary to eliminate these drawbacks, for example, by incorporating carbon nanoparticles into their structure. Selecting the right components of nanocomposites and improving the methods of their production are important elements for enabling technological development [29,33,34,35,36]. So far, no composite has been developed that meets most of the criteria listed above. There are constant attempts to find such a combination of materials that, with the right parameters, would also be biodegradable, and the cost of its production would be low enough to make it worthwhile to use on an industrial scale.

Having the above in mind, an attempt was made to design nanocomposites based on sodium alginate and chitosan (biodegradable polysaccharides with low acquisition costs), reinforced with graphene oxide and carbon nanotubes, and to characterize the obtained structures in terms of physicochemical and storage properties. The use of a simple, low-cost and environmentally friendly method of synthesizing nanocomposites is an additional advantage.

## 2. Materials and Methods

### 2.1. Materials

Chitosan from shrimp shells (high molecular weight: 310,000–375,000 Da, degree of deacetylation > 75%, (Sigma-Aldrich, St. Louis, MO, USA), sodium alginate (medium viscosity, Sigma-Aldrich, Poznan, Poland), anhydrous glycerine (pure for analysis, EuroChem BGD, Tarnów, Poland), carbon nanotubes 0.1% water suspension and graphene oxide 0.5% water suspension (Nanomaterials LS Co., Warsaw, Poland), lactic acid (80% water solution, pure for analysis, Pol-Aura, Morąg, Poland) and deionized water (demineralizer Polwater DL3-150, Labopol-Polwater, Kraków, Poland) were obtained.

### 2.2. Synthesis of Nanocomposites

#### 2.2.1. Preparation of 2% Alginate Gel

An amount of 10 g of alginate was measured and made up to 500 g with deionized water. The solution was stirred using a magnetic stirrer (Heidolph MR Hei-Tec, Schwabach, Germany) at 36 °C at 400–500 rpm until a homogeneous suspension was obtained. Then, 7.5 g of glycerine acting as a plasticizer was added and stirred again. In order to get rid of air bubbles, the mixture was put into an ultrasonic bath (POLSONIC, Warsaw, Poland).

#### 2.2.2. Preparation of 2% Chitosan Gel

An amount of 5 g of chitosan was dissolved in 245 g of a 0.1% lactic acid water solution. It was gelled using a magnetic stirrer (Heidolph MR Hei-Tec, Schwabach, Germany) at 500–700 rpm, heating the mixture to 75 °C. Then, 7.5 g of glycerine was spread in the gel and ultrasonicated.

#### 2.2.3. Synthesis of the Chitosan/Alginate Composite

Obtained alginate gel was mixed with chitosan gel in a 2:1 ratio, using a magnetic stirrer (Heidolph MR Hei-Tec, Schwabach, Germany) and a mechanical stirrer (Heidolph RZR2020, Schwabach, Germany). Approximately 200 g of gel was poured onto a metal tray and allowed to dry, thus obtaining a control film. The remainder of the gel was used to make nanocomposites.

#### 2.2.4. Synthesis of the Nanocomposites Containing Graphene Oxide

Approximately 200 g of the chitosan/alginate composite was mixed intensively with a mechanical stirrer (Heidolph RZR2020, Schwabach, Germany), slowly dropping in 8 g of 0.1% graphene oxide water suspension. Then, for better dispersion of nanoparticles in the matrix and to get rid of air bubbles, the gel was subjected successively to a homogenizer (Kinematica AG Polytron PT 2500 E, Malters, Switzerland) and an ultrasonic bath (POL-SONIC, Warsaw, Poland). Finally, it was spread evenly on a tray and dried at room temperature.

#### 2.2.5. Synthesis of the Nanocomposites Containing Carbon Nanotubes

An amount of 8 g of 0.1% carbon nanotube water suspension was added dropwise to 200 g of composite placed on a mechanical stirrer (Heidolph RZR2020, Schwabach, Germany). The resulting gel was stirred, homogenized and treated with ultrasound until the nanoparticles were evenly distributed. The nanocomposite was transferred quantitatively to a tray and allowed to dry.

**Scheme 1:**
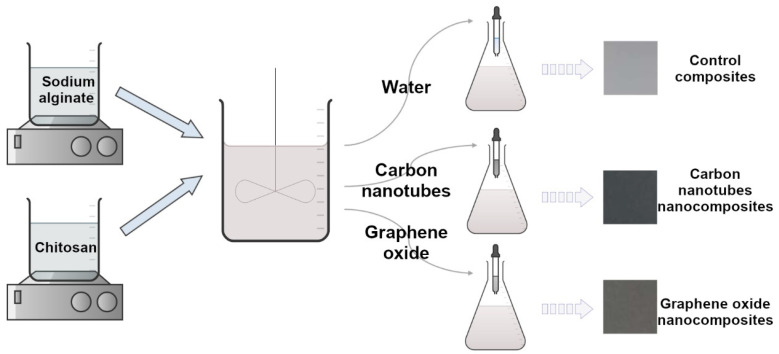
Visualization of the procedure for nanocomposite preparation.

A visualization of the nanocomposite preparation procedure is shown in the scheme below (Scheme 1).

### 2.3. Scanning Electron Microscopy (SEM)

The images were taken with a JEOL JSM-7500F high-resolution scanning electron microscope (Akishima, Tokyo, Japan).

### 2.4. Surface Color Analysis

The surface color was measured with a Konica MINOLTA CM-3500d (Konica Minolta Inc., Tokyo, Japan) with a 30 mm diameter measuring window, at illuminance and observer: D65/10°. The results were expressed using the CIE-Lab system. The following parameters were determined: L* (L* = 0 black, L* = 100 white); a*—the proportion of green (a* < 0) or red (a* > 0); and b*—the proportion of blue (b* < 0) or yellow (b* > 0). Measurements were made on a white background of the standard [29]. The experiment was repeated 5 times.

### 2.5. Thickness Measurement of Composites

The thickness was measured using a micrometer screw (Sylvac SA, Crissier, Switzerland) with an accuracy of 0.001 mm. Each film was measured five times, and the result obtained was the arithmetic average of all measurements.

### 2.6. Mechanical Properties of Composites

Mechanical investigations of the films were performed according to previous methodology [36]. The analyses were performed using a TA-XT plus texturmeter (Stable Micro Systems, Haslemere, UK). Films with dimensions of 35 mm × 6 mm were cut. The tensile test was performed at a speed of 2 mm/s. The maximum force required to break the sample and the relative elongation of the sample were determined. The measurements were performed in 10 repetitions. 

### 2.7. Water Content, Solubility and Swelling Degree of Nanocomposites

Composites were cut into a rectangle specimen (2 cm × 2 cm) and weighed, obtaining the initial weight of the sample (M1). Then, specimens were dried at 70 °C in an oven for 24 h, and the initial dry mass (M2) was then analyzed gravimetrically. The composites were dissolved in 30 mL of distilled water for 24 h at 25 °C. Afterwards, the specimens were superficially dried using filter paper and weighed (M3). The residual film samples were dried at 70 °C for 24 h in an oven, and the final dry mass was determined (M4). The analysis was performed in 3 replicates. The water content, solubility and degree of swelling of the composites were calculated according to the following equations [37]:

Water content (%) = (M1 − M2)/M1 × 100(1)

Solubility (%) = (M2 − M4)/M2 × 100 (2)

Swelling degree (%) = (M3 − M2)/M2 × 100(3)

### 2.8. Fourier-Transform Infrared Spectroscopy (FTIR)

The spectra of the composites were obtained using a Mattson 3000 FT-IR spectrophotometer (Madison, WI, USA), with a built-in 30-degree reflection cap ReFractance 30SPEC, with a MIRacle ATR accessory from PIKE Technologies Inc. The measurement was performed in the baseline infrared region, i.e., 4000–750 cm^−1^, with a resolution of 4 cm.

### 2.9. Ultraviolet and Visible Light Absorption Spectroscopy (UV-VIS)

Absorbance was measured using a Shimadzu 2101 spectrophotometer (Shimadzu, Kyoto, Japan), and measurements were made in the 200–800 nm wavelength range using 10 mL cuvettes.

### 2.10. Contact Angle and Surface Free Energy

Contact angle and surface free energy were measured using the Kruss Drop Shape Analyser DSA100M (Gmbh, Hamburg, Germany) equipped with a digital camera and environmental cell. The surface free energy was analyzed with the Owens–Wendt method [38,39], precisely as in our previous publications [27]. In this method, two liquids, bipolar water and polar diiodomethane, are used to determine the surface free energy of the examined materials. This methodology is generally accepted as the best for polymer substance evaluations. The test chamber temperature was controlled using a thermostatic water bath, allowing a constant temperature (22 ± 0.3 °C) and humidity. For each sample, at least five successive measurements were carried out.

### 2.11. Particle Size Analysis

The zeta potential and size of nanoparticles were measured via dynamic light scattering [40] with the Malvern Nano ZS instrument (Malvern Instruments Ltd., Worcestershire, UK), as described earlier [17,27,41]. Each measurement was repeated three times. The average zeta potential measurement error (standard deviation) was 5 mV maximum.

### 2.12. Statistical Analysis

The program Statistica 9.0 (Statsoft, Tulsa, OK, USA) was used to perform a one-way analysis of variance. The significance of differences was shown using Duncan’s test at a significance level of *p* = 0.05.

## 3. Results and Discussion

### 3.1. Scanning Electron Microscopy (SEM)

**Figure 1:**
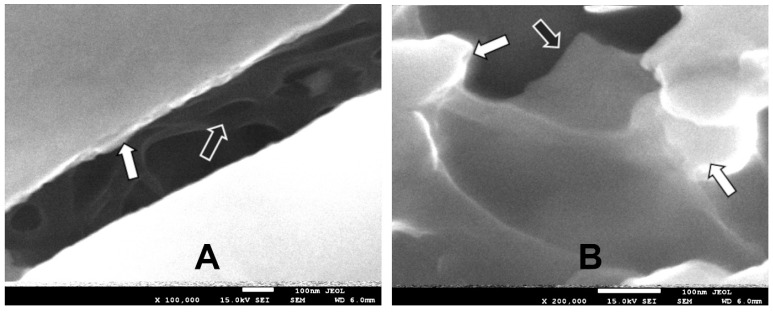
SEM images of a nanocomposite containing graphene oxide. White arrows indicate polysaccharide layers, and black arrows indicate nanoparticles. The images were taken at magnifications of 100,000× (**A**) and 200,000× (**B**).

Scanning electron microscopy was performed to visualize the surface of the fabricated nanocomposites and to show the size of the nanoparticles and the degree of their dispersion in the polysaccharide matrix. The graphene-oxide-modified chitosan/alginate composite is shown in Figure 1.

Passing an electron beam through the sample during the imaging process resulted in the formation of numerous cracks on the surface of the film. This made it possible to observe the layered structure of the graphene building up in the interior of the nanocomposite. In both images, we can see that the graphene sheets were well distributed in the matrix. This is very important because graphene oxide nanosheets, due to the strong forces of attraction between the planes, have a high tendency to aggregate, which makes them lose many beneficial properties. Their good dispersion in the composite material may have been due to the formation of hydrogen bonds and electrostatic interactions between the negatively charged oxygen groups of graphene oxide and the cationic groups of chitosan, which did not react with the alginate [29,42].

**Figure 2:**
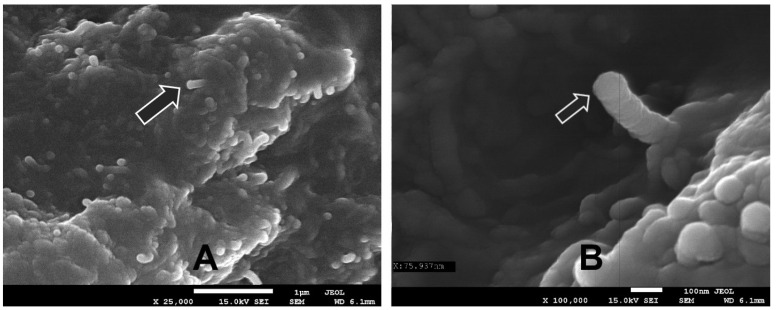
SEM image of a nanocomposite containing carbon nanotubes. Black arrows indicate nanoparticles. The images were taken at magnifications of 25,000× (**A**) and 10,000× (**B**).

**Figure 3:**
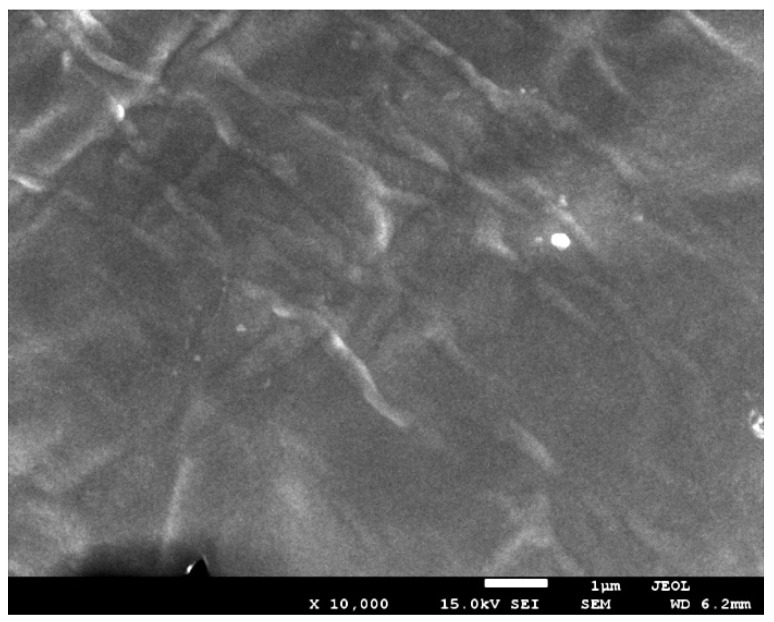
SEM image of a nanocomposite containing carbon nanotubes at 10,000× magnification.

The morphology of the films containing carbon nanotubes is shown in Figure 2 and Figure 3. In contrast to the graphene oxide nanocomposite, the surface structure of the film is not smooth but shows numerous bends due to the presence of nanotubes. The measurement of the diameter of the nanoparticle uncoated with the composite film yielded a result of 75,937 nm. Aggregates of nanotubes were observed located on the surface of the composite, visible in Figure 2. However, already deep within the matrix, the carbon nanostructures were uniformly dispersed (Figure 3).

### 3.2. Surface Color Analysis of Nanocomposites

Table 1 shows the color parameters of the nanocomposite surfaces. The control composites (chitosan/alginate composite) were characterized by high brightness, as evidenced by the value of the L* component at 95.53. The addition of graphene oxide and carbon nanotubes to the matrix significantly reduced the discussed result, affecting the darkening of the sample. The obtained values for a* > 0 showed a clear dominance of the green color in the case of the control composites, whereas the predominance of the red color was noticeable in the other samples. In all the composites produced, the proportion of the yellow color dominated over blue. This was particularly evident in the case of the composites containing no nanoparticles (b* > 0). The color and transparency of composites for the production of films and coatings are very important parameters, especially in those cases where the consumer’s choice of a particular product is determined by its visibility in the packaging. It should be noted, however, that this type of packaging is not suitable for products that require protection from external factors, mainly UV radiation. Such products include foods rich in fatty acids [5]. When designing packaging, it is necessary to keep in mind its intended use, and many variables must be taken into account, including the type and composition of the product, its shelf life, its texture and the storage time.

### 3.3. Thickness and Mechanical Properties of Nanocomposites

The thickness of the obtained films and their mechanical properties are shown in Table 2. As can be seen from the data obtained, the values were in the range of 0.057–0.080 mm. The control film had the highest thickness, and the other films were much thinner than it. The addition of graphene oxide and carbon nanotubes to the polysaccharide matrix improved its mechanical properties. In both cases, it recorded a significant increase in the breaking strength and an increase in the relative elongation of the nanocomposites. Thus, the data presented confirm the enhancement of the chitosan/alginate structure by 38% when carbon nanotubes are present in the structure, and by 70% when graphene oxide nanoparticles are present. In turn, the stretchability of the composites were enhanced by 29% and 27%, respectively. Such a significant improvement in mechanical properties is due to a properly conducted synthesis process, by which the carbon nanostructures were uniformly dispersed in the polysaccharide matrix. The addition of carbon nanoparticles strengthened the structure of the polysaccharides, and the TS values obtained were higher than those for the low-density polyethylene (LDPE). In contrast, the extensibility of carbon nanocomposites was slightly higher than polyester (PE) and polyvinylidene chloride (PVDC) [43].

### 3.4. Water Content, Solubility and Degree of Swelling of Nanocomposites

The water content in the control composites and nanocomposites did not vary, but statistically significant differences were observed between nanocomposites (Table 3).

The incorporation of carbon nanoparticles into the composite structure significantly reduced the solubility of the nanocomposites compared to the control (by 14%). It may be a desirable feature when looking for systems with increased barrier properties [29]. The presented values were higher than those in our earlier work for nanocomposites, which consisted of potato starch/chitosan gels with the GO [29]. It should be noted, however, that in the cited example, both the proportions of polysaccharides and carbon nanoparticles used were different than those in the present work. Moreover, the method of synthesizing the nanocomposites varied significantly. The solubility of a nanocomponent is an essential feature which determines its further use/purpose. Nanocomposites with high solubility can be used as coatings, films or carriers, where the speed and degree of the release of the package contents into another medium play a key role. Therefore, it can be important in biomedical applications. It is also worth mentioning that the increased solubility of the nanocomposite can reduce the decomposition time of the material in the environment, thus increasing its biodegradability. The low solubility of nanocomposites is a desirable feature in the storage of products with high water content, which may result in the penetration of components of such materials into food or drugs [29].

No statistically significant differences were observed in the degree of swelling of the samples; thus, the introduction of graphene oxide and carbon nanotubes into the system did not block some of the active groups for water absorption [29].

### 3.5. Fourier-Transform Infrared Spectroscopy (FTIR)

**Figure 4:**
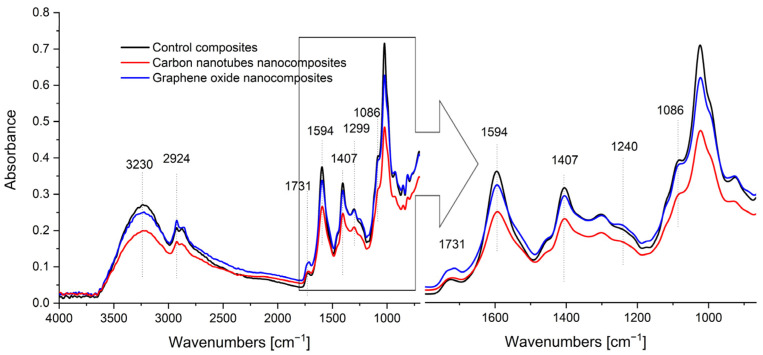
FTIR spectra of composites: control composites, carbon nanotube nanocomposites and graphene oxide nanocomposites.

Using infrared spectroscopy, the types of groupings present in the resulting composites were indicated. This made it possible to determine whether the introduction of graphene oxide and carbon nanotubes into the polysaccharide composites caused significant changes in the chemical structure of the films (Figure 4).

The broad absorption bands in the 3000–3500 cm^−1^ range are associated with the presence of hydrogen-bonded hydroxyl (-OH) and amine (-NH_2_) groups. The bands at 2924 cm^−1^ and 2872 cm^−1^ are characteristic of saturated hydrocarbons. They can be attributed successively to asymmetric and symmetric stretching vibrations of C-H groups. Peaks in the 1500–2000 cm^−1^ range correspond to groups containing carbon atoms and double bonds in their structure. The peaks present on the graph near 1731 cm^−1^ indicate the presence of C=O groups. They can be conjugated to each other or to C=C bonds, the presence of which is indicated by peaks located in the 1600–1870 cm^−1^ region. The peak characteristic of the chitosan/alginate complex at a wave number of about 1594 cm^−1^ and the intense peaks located at about 1086 cm^−1^ report the presence of deformational N-H symmetric vibrations contained in the -NH_3_^+^ ion. They indicate the formation of ionic bonds between the negatively charged carboxyl groups belonging to alginate and the positively charged amino groups of chitosan (~NH_3_^+^ —.... -O(O)C~). In the dactyloscopic range (below 1500 cm^−1^), which has an arrangement of bands characteristic of the molecule, a wave number close to 819 cm^−1^ can also be seen, caused by Na-O bonds occurring in the β-d-mannuronic acid and α-l-guluronic acid residues of alginate [17,24,44].

No significant differences were observed between the spectra of the nanocomposites and those of the chitosan/alginate composite. The lack of band shifts means that the introduction of graphene oxide or carbon nanotubes into the polysaccharide matrix did not significantly change the molecular interactions occurring in it. We observed a slight shift and a change in the intensity of the band at 1731 cm^−1^, which may have been caused by the formation of hydrogen bonds between the carboxylic and hydroxyl groups of the composite components [45], and a slight decrease in absorbance intensity after modification may have been due to the formation of new hydrogen bonds and differences in water content [29,42], which correlates with the water content results (Table 3).

### 3.6. Ultraviolet and Visible Light Absorption Spectroscopy (UV-VIS)

**Figure 5:**
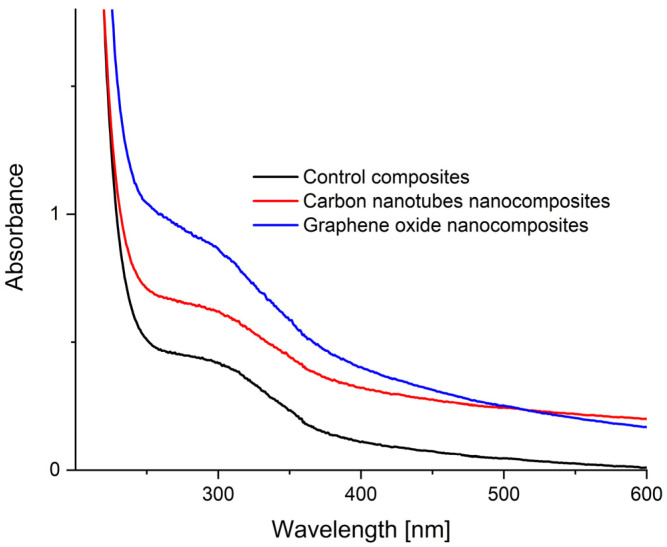
UV-VIS spectra of composites: control composites, carbon nanotubes nanocomposites and graphene oxide nanocomposites.

The UV-VIS spectra of the resulting nanocomposites, recorded using a UV-VIS spectroscope, are shown in Figure 5.

Absorption maxima occurred at wavelengths close to 230 nm. In the 250–370 nm range, broad bands were observed, which were indicative of different sizes of dispersed nanoparticles. All peaks noted were in the near-ultraviolet region (200–400 nm). This means that they came from the transitions of binding electrons responsible for unsaturated bonds to anti-binding orbitals (π → π*) and from the excitation of non-binding electrons (n → π*). The unsaturated bonds most likely originated from the carbon–carbon (C=C) or carbon–oxygen (C=O) groupings present in the nanocomposite structures, and the non-bonding electrons were present in the oxygen atoms. For a composite containing graphene oxide, the absorption maximum was shifted toward longer wavelengths, which may mean that the compound contained more conjugated bonds. In addition, films enriched with carbon nanoparticles presented higher absorbance than the polysaccharide composite, so they were more effective matrices in protecting against ultraviolet radiation [17,24].

### 3.7. Contact Angle and Surface Free Energy

As can be seen, the initial material (control composites, made of chitosan/alginate material, stabilized by glycerin) was characterized by only dispersion interactions (Table 4). The polar surface energy was practically undetectable there. Adding graphene and graphene particles with carbon nanotubes to the chitosan/alginate foil changed the material’s properties. The modified materials’ total surface free energy increased significantly due to the increase in the polar energy of the tested films. We observed a practical equilibrium between polar and dispersive energies in the obtained materials. In the case of the film doped with graphene oxide, we observed a slight advantage of polar interactions over dispersions. The introduction of carbon nanotubes slightly changed the parameters of the material. In this situation, field energy slightly dominated over dispersion energy.

### 3.8. Particle Size Analysis

Measurements of the size and zeta potentials proved that the introduction of graphene and CNT to the material caused a multiple increase in the size of biopolymer aggregates and a decrease in their surface zeta potential from about −1.2 mV to the value of −28.3 and −39.2 mV, respectively (Table 5). The increase in particle size could be caused by the wrapping of the polysaccharide chains (in the case of CNTs) or the formation of layered structures (in the case of GO) due to electrostatic interactions and hydrogen bonding [46,47]. The improvement in the mechanical properties of the nanocomposites compared to the control sample confirmed the results (Table 2).

## 4. Conclusions

This study confirmed the formation of carbon nanocomposites on a polysaccharide matrix. FTIR spectroscopy showed that the nanoparticles that we used did not interact chemically with the polymers and therefore did not disrupt their structure. The polysaccharides only acted as a matrix. Both scanning electron microscopy and ultraviolet and visible light absorption spectroscopy showed that the dispersed carbon structures had different sizes. Modification of the chitosan/alginate composite induced its significant darkening, increased the proportion of the color red and improved its mechanical properties. Ultraviolet and visible light absorption spectroscopy proved the positive effect of nanoparticles on the absorption capacity of the composite materials. The film containing graphene oxide showed the highest absorbance. They showed good barrier properties and can protect food from ultraviolet radiation. Based on the results obtained, it can be concluded that the enrichment of the polysaccharide composite with the discussed nanoparticles has a beneficial effect on its physicochemical and functional properties. The obtained nanocomposites show great application potential in many areas of science and industry, including the food sector as an alternative to packaging materials made from plastics.

## Figures and Tables

**Table 1:** Surface color parameters of nanocomposites.

Sample	L*	a*	b*
Control composites (chitosan/alginate)	95.53 ± 0.04 ^a^	−0.72 ± 0.01 ^c^	7.06 ± 0.09 ^b^
Carbon nanotube nanocomposites	57.74 ± 0.40 ^c^	0.33 ± 0.02 ^b^	6.53 ± 0.02 ^c^
Graphene oxide nanocomposites	67.18 ± 0.65 ^b^	2.61 ± 0.12 ^a^	16.30 ± 0.16 ^a^

The parameters in columns with the same letters (a, b or c) do not differ statistically.

**Table 2:** Thickness and mechanical parameters of the nanocomposites.

Sample	Thickness (mm)	Tensile Strength (MPa)	Elongation at Break (%)
Control composites (chitosan/alginate)	0.080 ± 0.003 ^a^	17.5 ± 0.9 ^b^	16.9 ± 1.6 ^b^
Carbon nanotubes nanocomposites	0.058 ± 0.005 ^b^	24.2 ± 3.0 ^a^	21.8 ± 3.1 ^a^
Graphene oxide nanocomposites	0.061 ± 0.009 ^b^	29.9 ± 4.2 ^a^	21.5 ± 2.9 ^a^

The parameters in columns with the same letters (a or b) do not differ statistically.

**Table 3:** Water content, solubility and degree of swelling of composites.

Sample	Water Content (%)	Solubility (%)	Swelling Degree (%)
Control composites (chitosan/alginate)	11.92 ± 2.25 ^ab^	61.84 ± 6.54 ^a^	94.61 ± 0.81 ^a^
Carbon nanotube nanocomposites	10.64 ± 0.51 ^b^	53.17 ± 0.99 ^b^	94.03 ± 0.50 ^a^
Graphene oxide nanocomposites	13.55 ± 0.42 ^a^	53.14 ± 0.51 ^b^	94.05 ± 0.28 ^a^

The parameters in columns with the same letters (a or b) do not differ statistically.

**Table 4:** Surface color parameters of nanocomposites.

Sample	Contact Angle [°]		Surface Free Energy [mJ/m^2^]		
	Water	Diiodomethane	Dispersive	Polar	Total Free Energy
Control composites (chitosan/alginate)	90.00	49.66	37.30	1.23	38.53
Carbon nanotube nanocomposites	42.60	42.45	29.99	27.70	57.76
Graphene oxide nanocomposites	40.64	50.90	24.55	32.80	57.34

**Table 5:** Particle size and zeta potential of nanocomposites.

Sample	Zeta Potential [mV]	Size [nm]
Control composites (chitosan/alginate)	−1.2	8000
Carbon nanotube nanocomposites	−28.3	>20,000
Graphene oxide nanocomposites	−39.2	20,000

## Data Availability

The data presented in this study are available on request from the corresponding author.
